# Supplementary figures and images for: The anti-neural role of BMP signaling is a consequence of its ancestral function in dorsoventral patterning
Source: PLoS Biol. 2026 May 21;24(5):e3003803. doi: 10.1371/journal.pbio.3003803 (PMC13193452; doi:10.1371/journal.pbio.3003803)

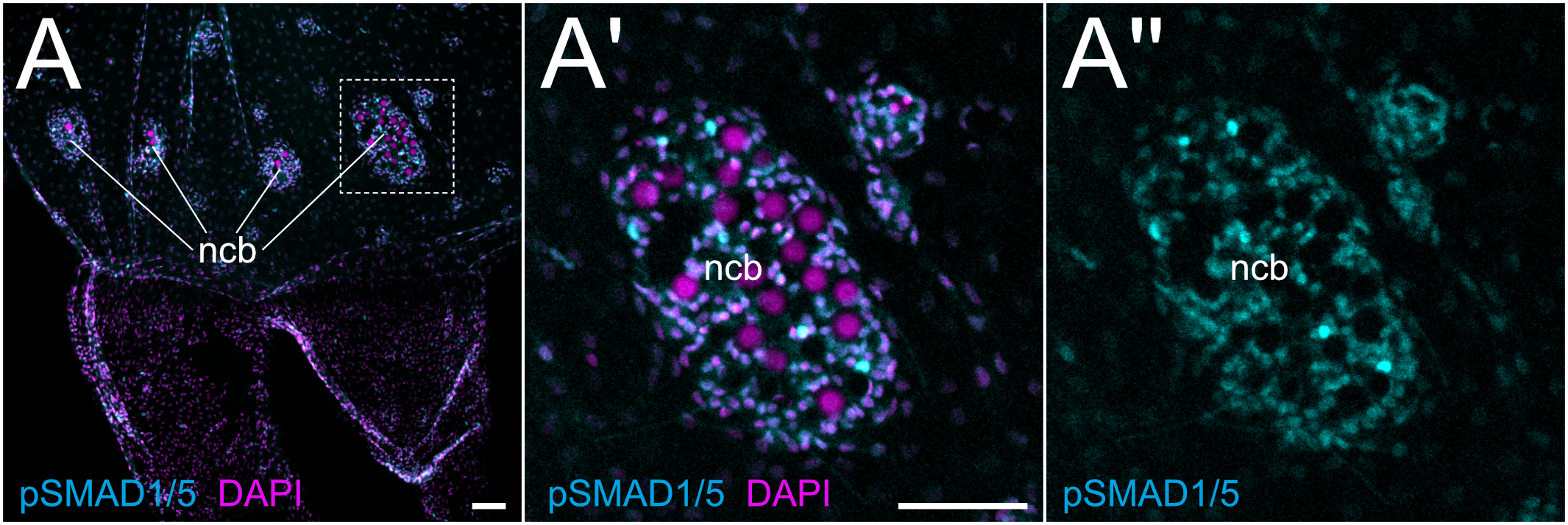

Supplement: S1 Fig — ncb - nematocyst battery, Scale bars 50μm. (TIFF) [file pbio.3003803.s001.tiff]

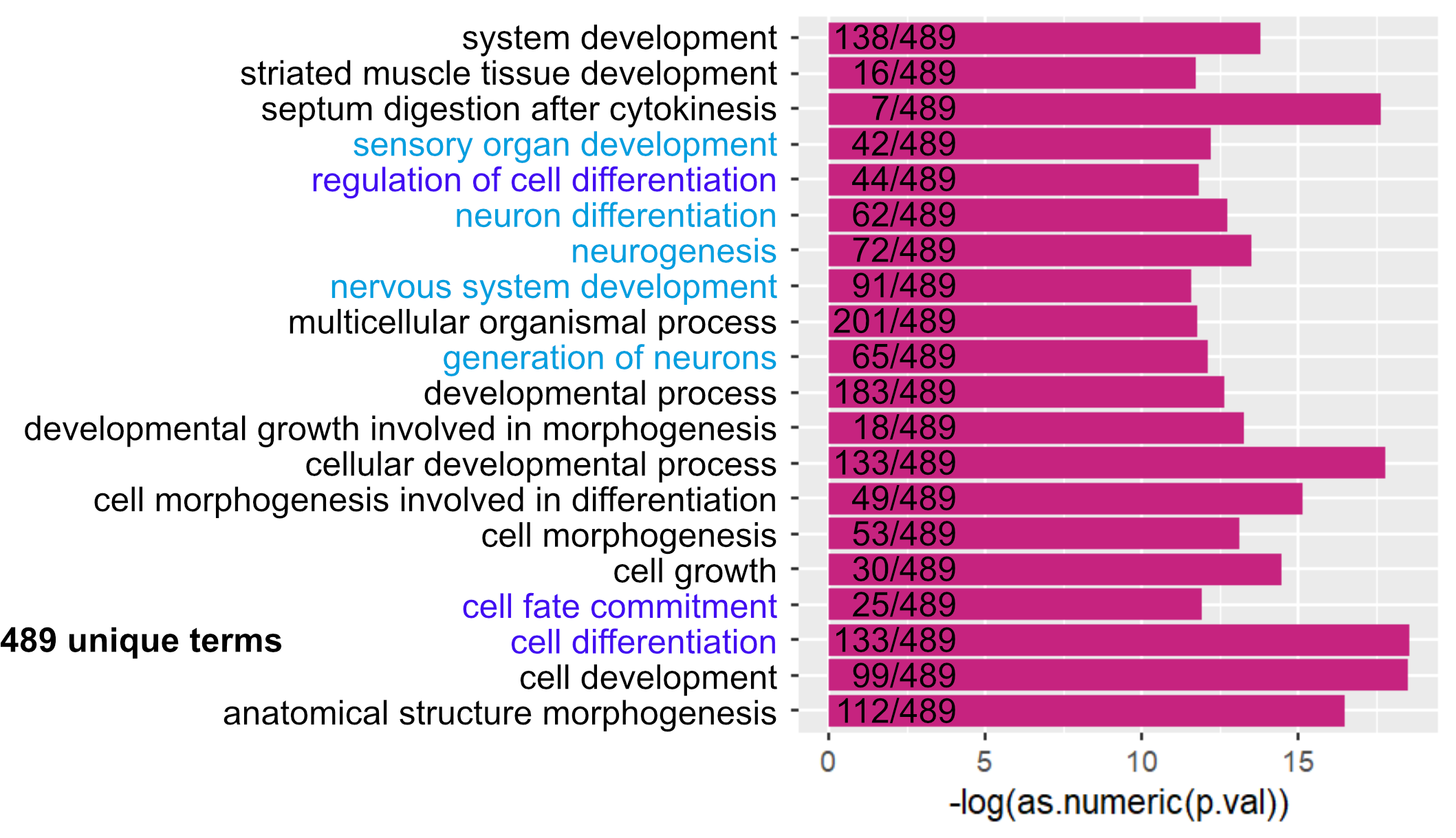

Supplement: S2 Fig — Numerical data for the bar plot can be found in the K02288_adj.GOenrich sheet of the S1 Table. (TIFF) [file pbio.3003803.s002.tiff]

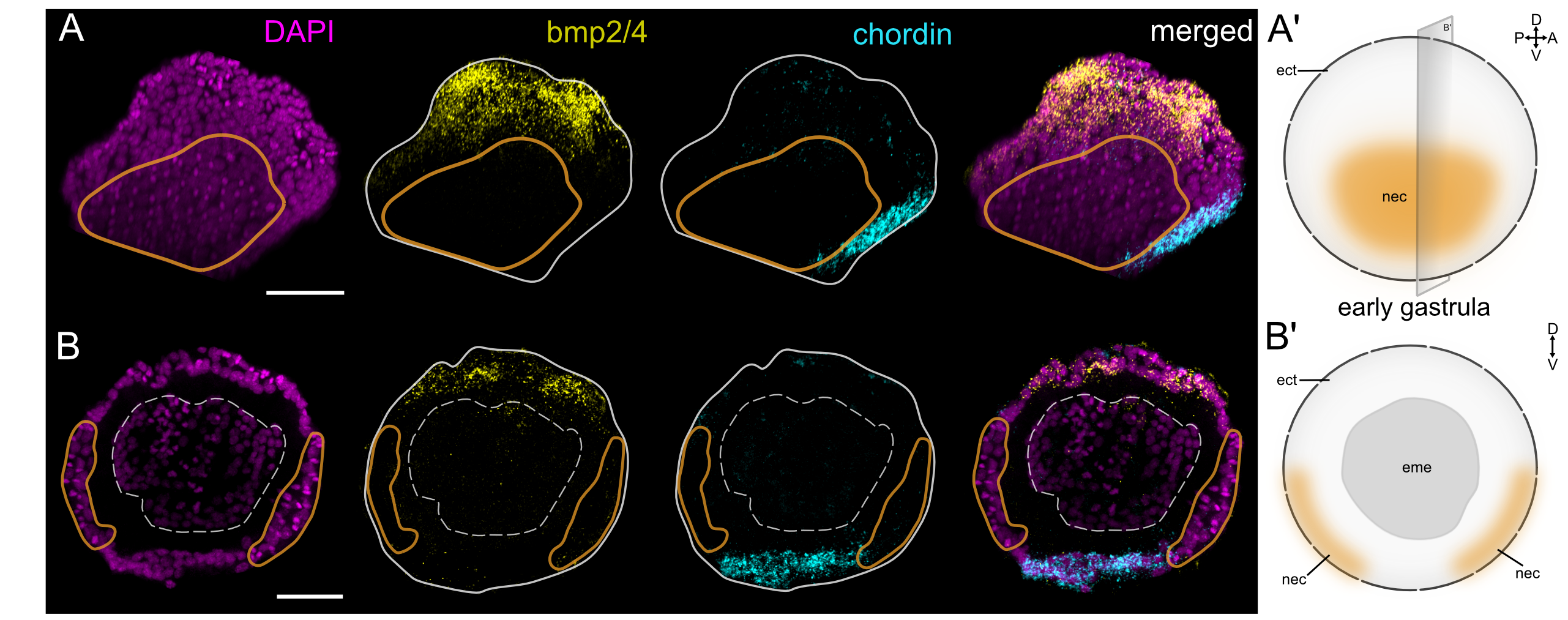

Supplement: S3 Fig — (A-A’) Lateral view and (B-B’) frontal view and the corresponding sketches of the anatomy of the embryo. Orange outlines demarcate the neuroectoderm (A, B) and dashed outlines demarcate the endoderm (B). Scale bars 50 μm. Abbreviations: ect, ectoderm; eme, endomesoderm; nec, neuroectoderm. (TIFF) [file pbio.3003803.s003.tiff]

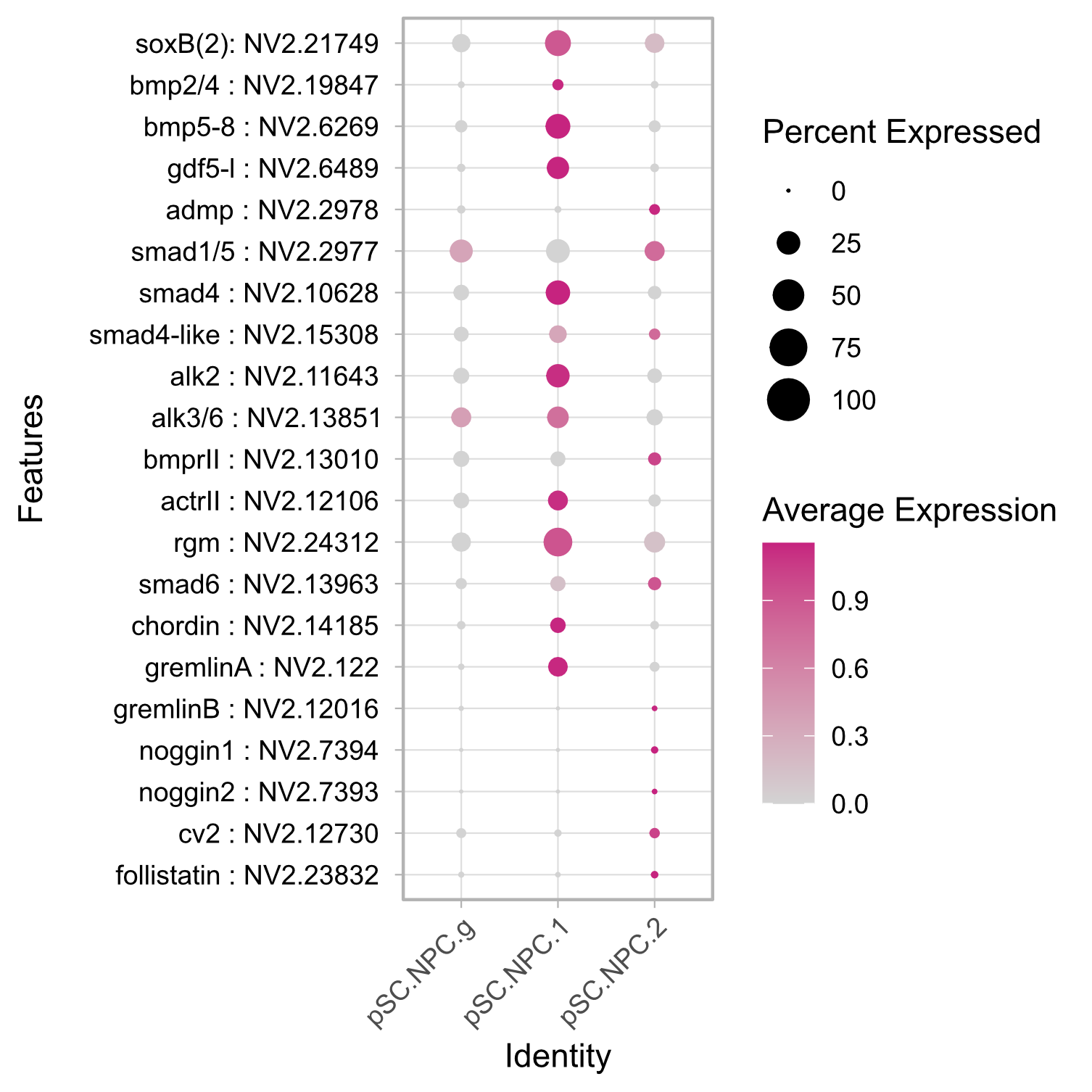

Supplement: S4 Fig — Average scaled expression of 0 or below is indicated in gray. Numerical data for the plot can be extracted by running the R scripts found here 10.5281/zenodo.19686897. (TIFF) [file pbio.3003803.s004.tiff]

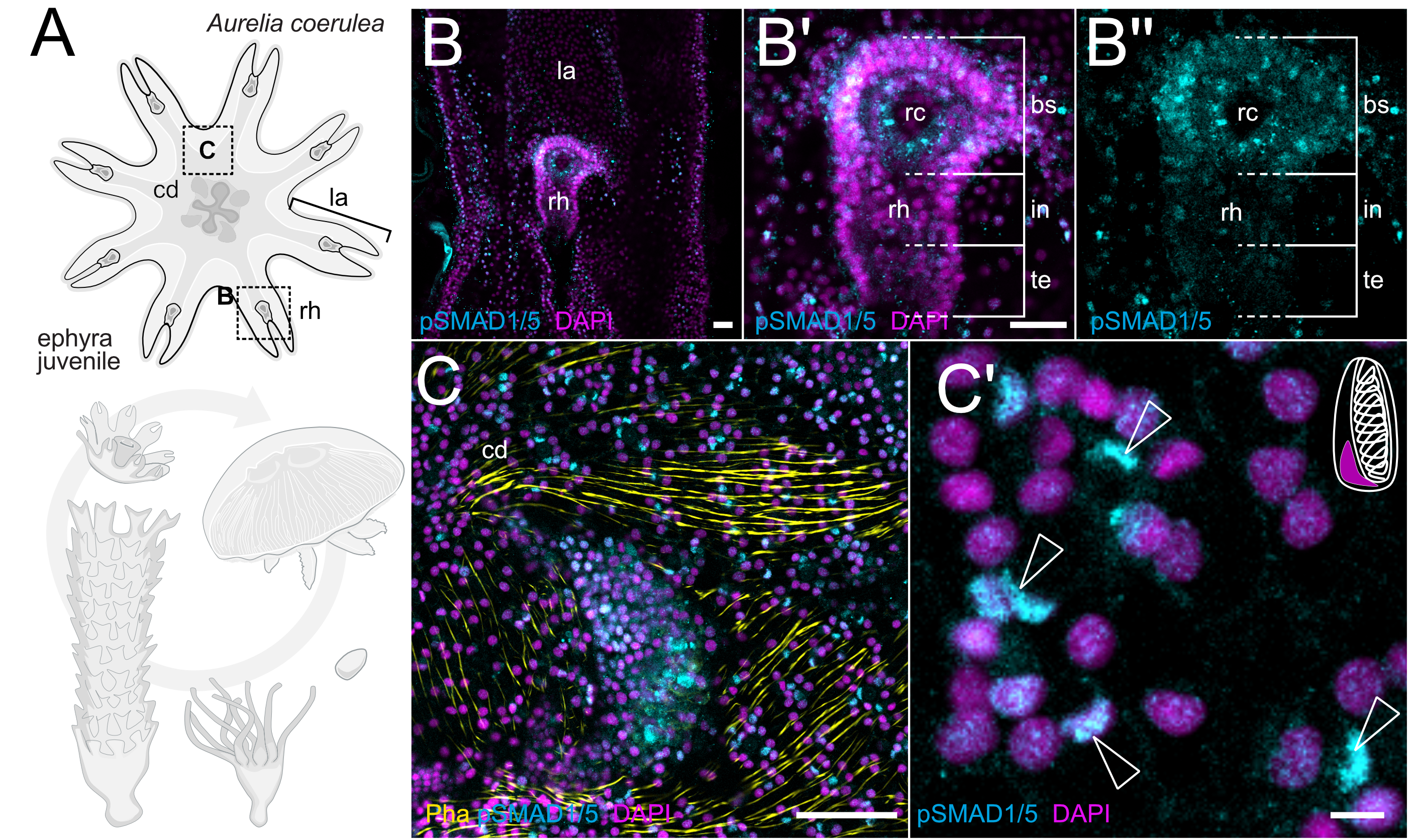

Supplement: S5 Fig — (A) Life cycle schematic of Aurelia coerulea, details of the ephyra juvenile stage. (B) Rhopalia in the lappets exhibit pSMAD1/5 staining at the rhopalic canal (rc). (C) pSMAD1/5 staining in the central disc of the ephyra. Pha - phalloidin. (C’) Detail showing pSMAD1/5-positive crescent-shaped nuclei typical for cnidocytes. cd - central disc, rh - rhopalium, rc - rhopalial canal, bs-basal segment, in-intermediate segment, te-terminal segment; Scale bar (B-B’) 25 μm, (C) 50 μm, (C’) 5 μm. (TIFF) [file pbio.3003803.s005.tiff]

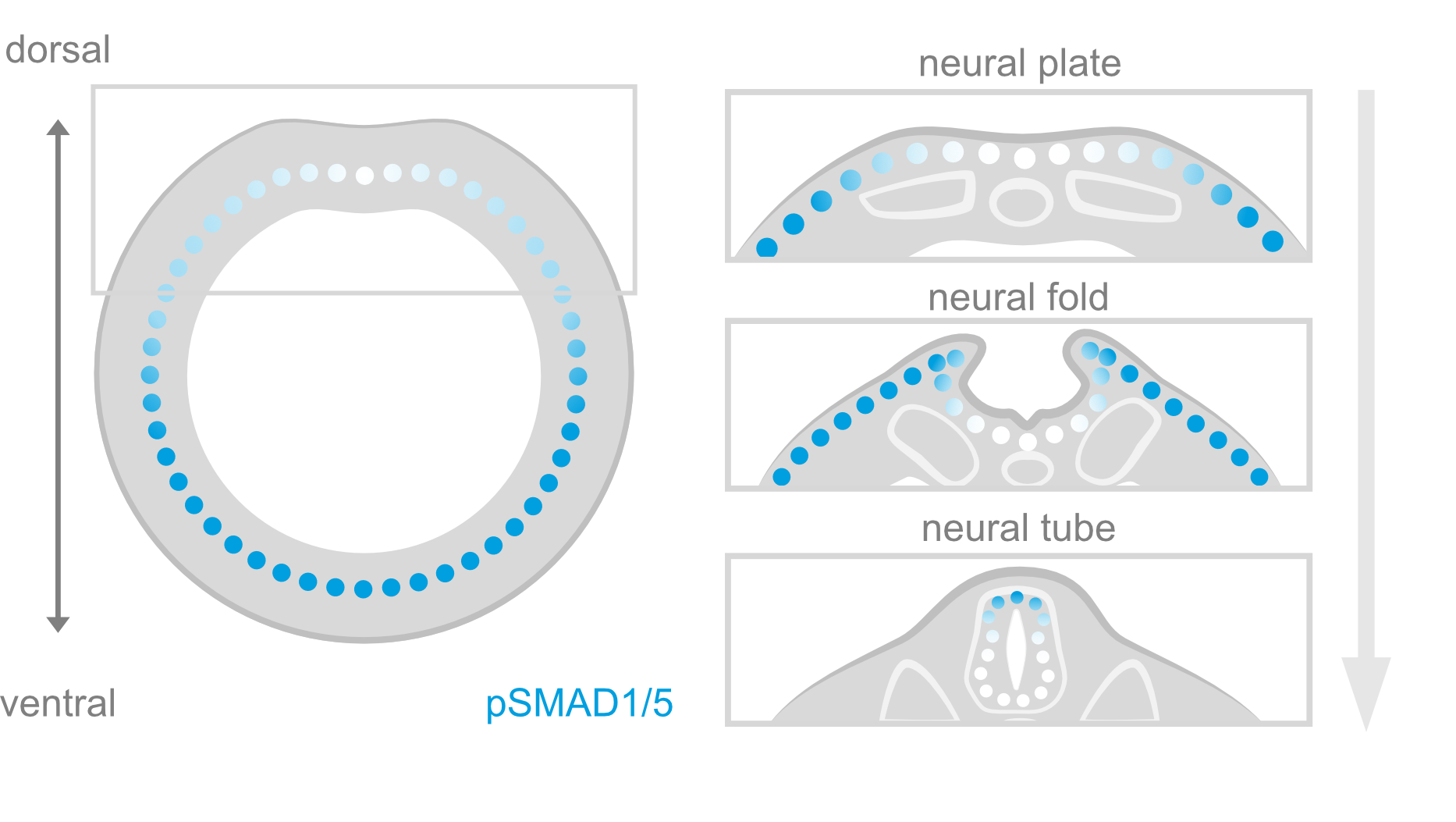

Supplement: S6 Fig — The dorsalmost part of the neural tube originates from the ventralmost neurectoderm. Blue circles indicate pSMAD1/5 positive nuclei. (TIFF) [file pbio.3003803.s006.tiff]

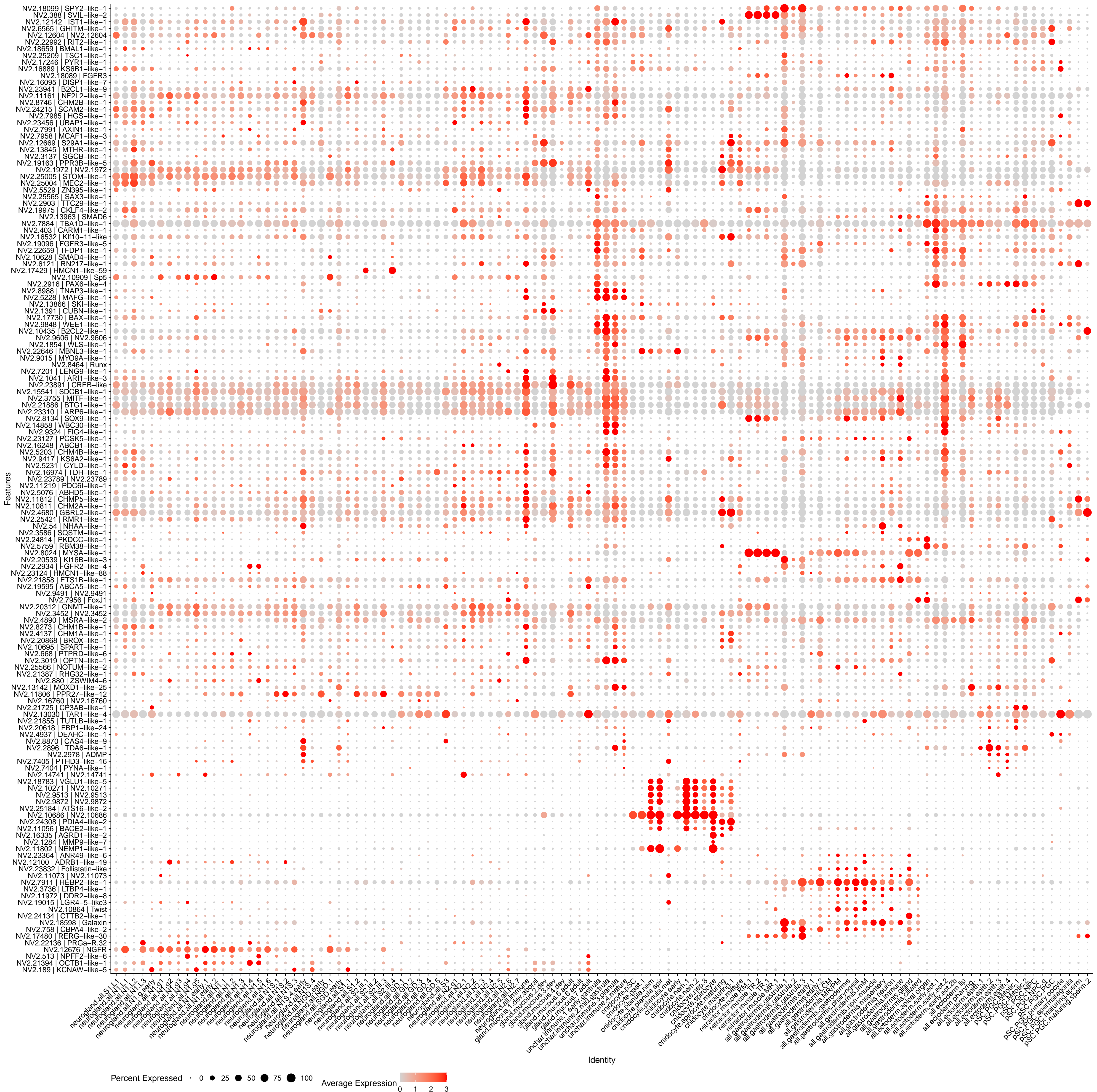

Supplement: S1 Plot — The R-object containing all the raw scRNA-Seq data used in this study is available at https://cells.ucsc.edu/sea-anemone-atlas/Nv2/all/AllData.Robj. The list of gene models and the R script “scRNA-SeqDotPlots_S1_Plot-S4_Plot.R” allowing to reproduce this plot and extract the underlying data can be found at 10.5281/zenodo.19686897. (PDF) [file pbio.3003803.s007.pdf]

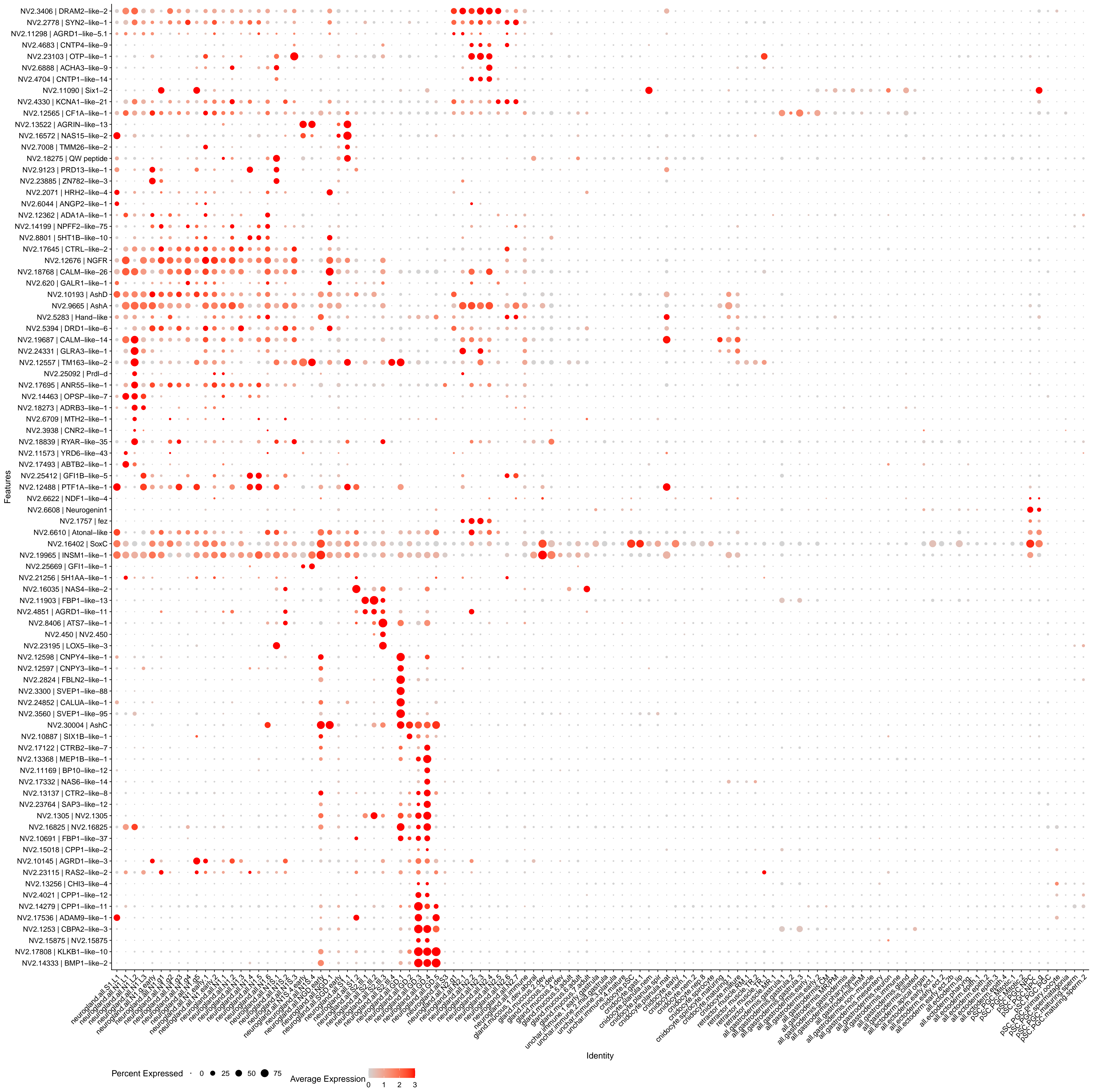

Supplement: S2 Plot — The R-object containing all the raw scRNA-Seq data used in this study is available at https://cells.ucsc.edu/sea-anemone-atlas/Nv2/all/AllData.Robj. The list of gene models and the R script “scRNA-SeqDotPlots_S1_Plot-S4_Plot.R” allowing to reproduce this plot and extract the underlying data can be found at 10.5281/zenodo.19686897). (PDF) [file pbio.3003803.s008.pdf]

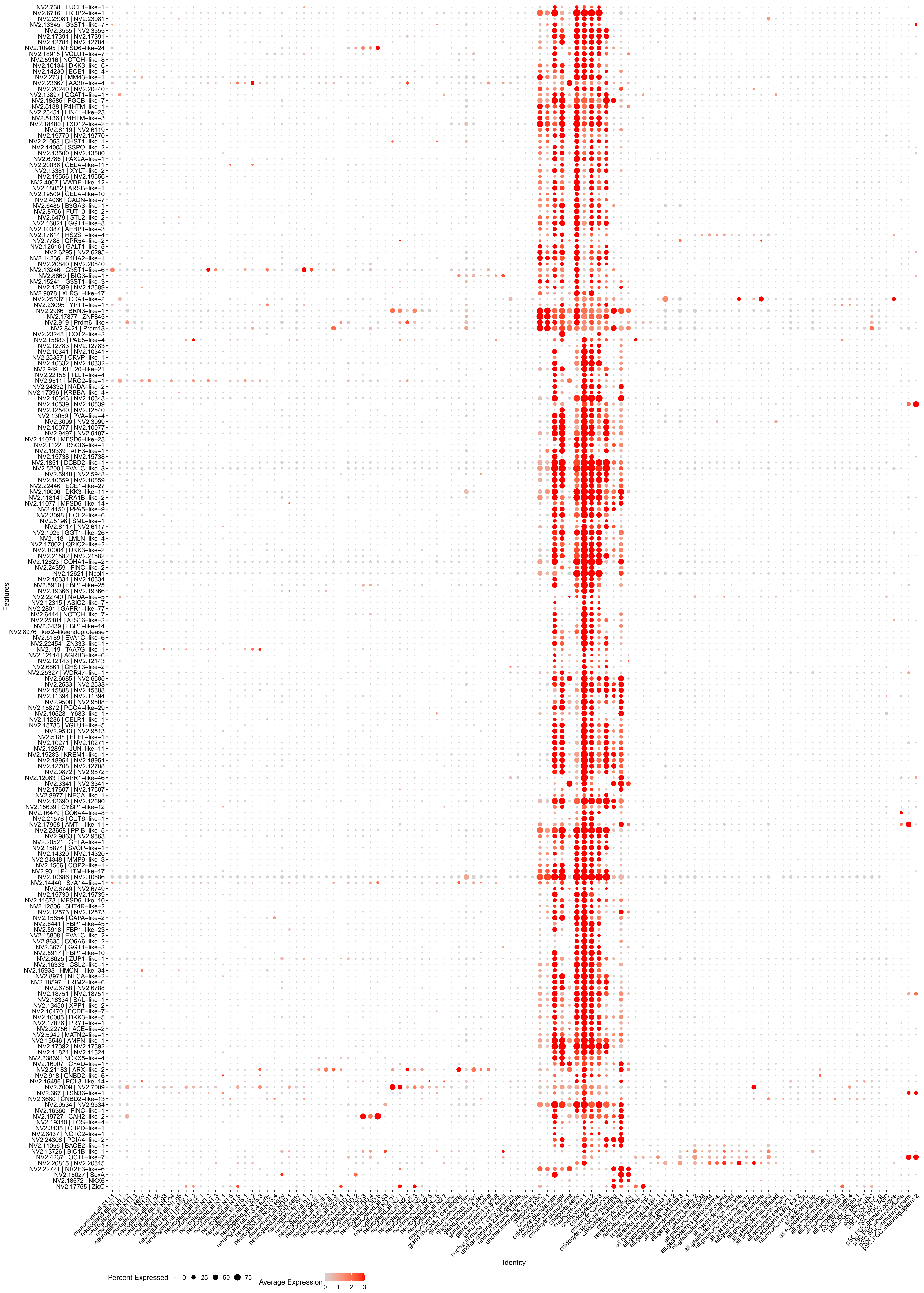

Supplement: S3 Plot — The R-object containing all the raw scRNA-Seq data used in this study is available at https://cells.ucsc.edu/sea-anemone-atlas/Nv2/all/AllData.Robj. The list of gene models and the R script “scRNA-SeqDotPlots_S1_Plot-S4_Plot.R” allowing to reproduce this plot and extract the underlying data can be found at 10.5281/zenodo.19686897). (PDF) [file pbio.3003803.s009.pdf]

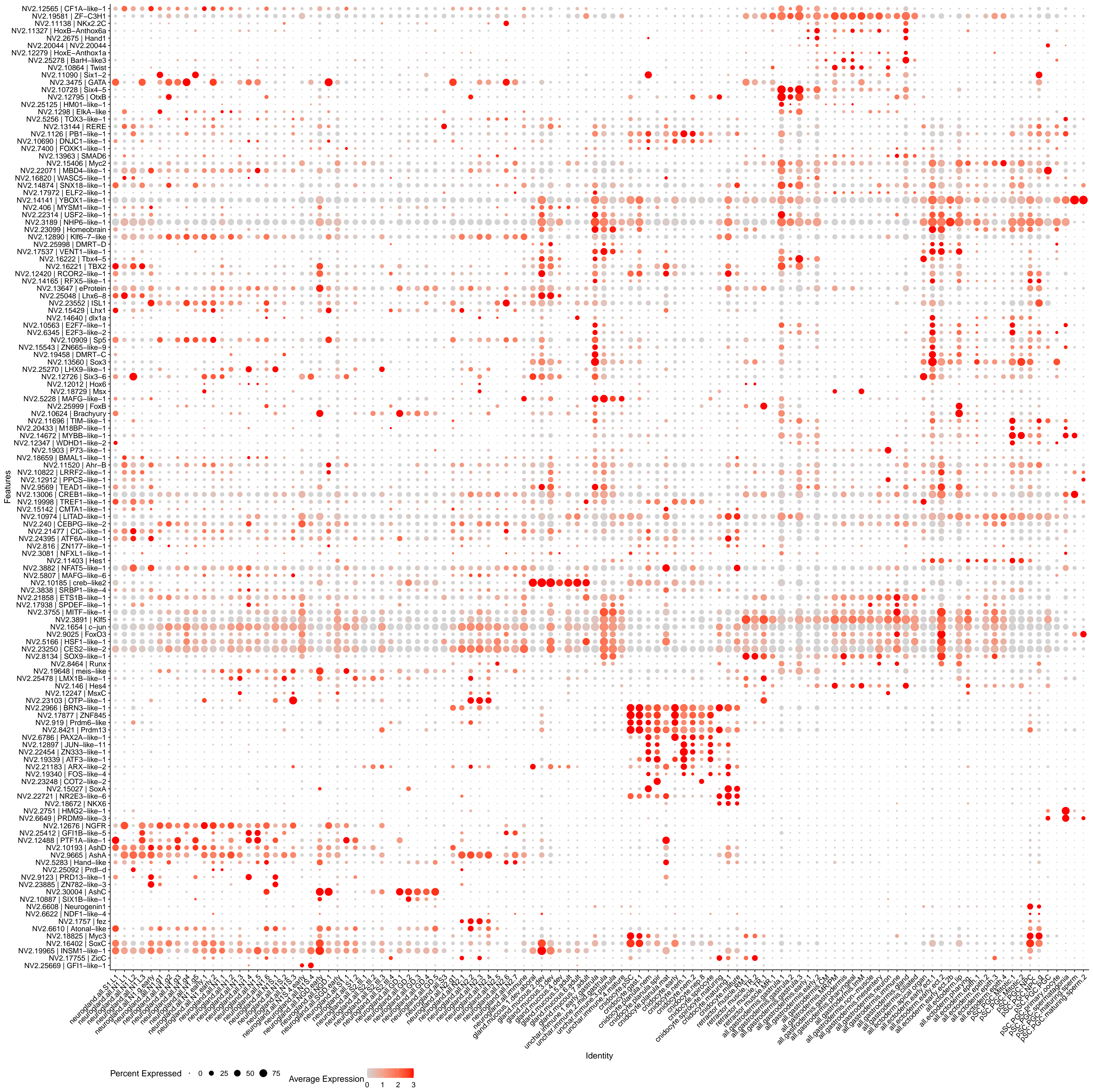

Supplement: S4 Plot — The R-object containing all the raw scRNA-Seq data used in this study is available at https://cells.ucsc.edu/sea-anemone-atlas/Nv2/all/AllData.Robj. The list of gene models and the R script “scRNA-SeqDotPlots_S1_Plot-S4_Plot.R” allowing to reproduce this plot and extract the underlying data can be found at 10.5281/zenodo.19686897). (PDF) [file pbio.3003803.s010.pdf]
