# Supplementary material for: The anti-neural role of BMP signaling is a consequence of its ancestral function in dorsoventral patterning
Source: PLoS Biol. 2026 May 21;24(5):e3003803. doi: 10.1371/journal.pbio.3003803 (PMC13193452; doi:10.1371/journal.pbio.3003803)
Supplement: Attachment — Submitted filename: Responses to reviewers.docx [file pbio.3003803.s016.docx]

**Reviewers' comments**

**Rev. 1:**

In this manuscript, the authors show that BMP signaling is active in the adult sea anemone Nematostella within particular neuronal cell types. Through functional experiments using BMP receptor inhibitor and RNA-seq analysis, they also demonstrate that BMP signaling can positively regulates neuronal gene expression and neurogenesis. They also show that BMP signaling is active in the medusozoan nervous system, suggesting it may function in promoting neurogenesis for both anthozoans and medusozoans. Finally, they show that BMP signaling is also active in the neurogenic domain of Chaetognath embryos, and later in the sensory organ of the juvenile. Based on these results, the authors suggested that BMP signaling can positively regulate neurogenesis in a wide variety of animals and may represent an ancestral feature, predating the cnidarian-bilaterian divergence. They also propose that the "anti-neural" function of BMP signaling in classical bilaterian models may represent the co-option of its global role in the dorsoventral patterning of the ectoderm.

In general, this manuscript is well written, and the data presented are mostly well presented and robust, especially for the parts on Nematostella. However, I do have some comments regarding the observation of BMP activity in medusozoan and Chaetognath, as well as on its activity in relation to neurogenesis in these animals. More specifically, the weakness of this manuscript is that the authors did not provide experimental evidence to support the "pro-neural" function of BMP signaling in medusozoan and Chaetognath. Therefore, while the results of pSMAD1/5 staining in these animals are quite striking, there remains a large degree of uncertainty about the conclusions of BMP activity for their neural development. It would become much more convincing if the pSMAD1/5 staining results in medusozoan and Chaetognath were also integrated by functional studies, such as the experiments applying BMP receptor inhibitor K02288 (like that conducted in Nematostella) or recombinant BMP

protein to elevate BMP signaling level (see Denes et al., Cell 2007, 129:277-88).

Response:

We agree in principle that a thorough investigation of the role of BMP signaling in the development of the chaetognath is of interest, however, they are out of scope for this paper. Spadella cephaloptera reproduces reliably only within a very short time window during the summer, and obtaining sufficient amounts of synchronously developing eggs for the proposed inhibitor experiments is an extremely challenging task. Currently, the Wollesen group has no funding available for them to return to Roscoff and repeat these experiments in the coming summer, and the contract of the PhD student, June Ordoñez, has already expired. Finally, a protocol enabling the delivery of inhibitors through the egg capsule surrounding the early embryo has not yet been established. So, the proposed experiments will have to be done in the future once Spadella becomes a better established model. On the other hand, the point we are making in the paper is not that BMP signalling is required for the neural development in Spadella but that Spadella central nervous system (or at least the dorsal half of it) is specified within the BMP signaling domain - so, BMP signaling in Spadella is not anti-neural.

We write:

“These results show that parts of the central nervous system of Spadella, particularly the dorsal regions of the neuroectoderm, are located within the pSMAD1/5-positive domain, and that BMP signaling remains active in the ventral nerve center and in the forming sensory organs later in development.”

In the revised version of the manuscript, we followed the Reviewer’s suggestion to perform functional experiments on a medusozoan. Since antibody staining with antibodies against neuronal markers did not work in Aurelia ephyra (see response to the next comment), we obtained another species, Stomolophus sp., from the Haus des Meeres aquarium in Vienna, in which antibody stainings proved to work. We treated 2-month old Stomolophus metaephyra with K02288 and performed RNA-Seq to see how putative neuronal genes were affected by BMP signaling inhibition. To our satisfaction, just like in Nematostella, they were predominantly suppressed, supporting the pro-neural function of BMP signaling. There is a new chapter, a new main text figure, and several supplementary data items describing this.

About the Aurelia ephyra data presented in Fig 8 and the text on page 17, it is uncertain to me whether the observed pSMAD1/5 signals in the rhopalic canal are really located in neuronal cells. Although the authors mentioned that they were unable to combine the staining of neuronal markers with pSMAD1/5 staining, why not using antibodies against neuropeptides (for example, see Nakanishi et al., Dev Genes Evol 2009, 219:301-17. doi: 10.1007/s00427-009-0291-y) to label neuronal cells in comparable samples separately and then compare the result with pSMAD1/5 staining to see whether they are showing similar pattern?

Response:

We thank the Reviewer for the suggestion. We tested several peptide antibodies in Aurelia ephyra but were unsuccessful. We also contacted Prof. Nakanishi and asked him for the details of the antibodies he used, but unfortunately they are not sold in the EU. Therefore, we obtained another scyphozoan species, Stomolophus sp. from the Vienna aquarium to perform co-stainings of pSMAD1/5 and neuronal markers. Here, we successfully tested anti-FMRF (Neuromics, RA20002;), anti-Serotonin/5-HT (Immunostar, CAT20080;), anti-GLWamide 1675 III p (Schmich et al., 1998) and anti-beta-Tubulin (Proteintech, 66240-1-Ig) that we summarized in Figure 9. Several other tested antibodies including anti-FMRF antibodies (Sigma, AB15348; and BIOMOL, FA1155;) and anti-HuC/HuD 16A11 (Invitrogen, A-21271;) produced no specific staining. There is a new Fig. 6 showing anti-pSMAD1/5 stainings and the abovementioned antibodies.

It is interesting to see that the dorsal expression of BMP2/4 and the ventral expression of Chordin seems to be consistent with the observed graded pSMAD1/5 signals in the developing Chaetognath embryo (Fig 9 and S1 File-Fig 2). It is not mentioned whether this early BMP signaling gradient might be involved in the specification and patterning of neuroectoderm in the ventral part of the Chaetognath gastrula. More specifically, is inhibition of BMP signaling necessary for the initial specification of ventral nervous system in Chaetognath embryo? This is an important question that needs to be address in the manuscript.

Response:

Performing inhibitor treatments on Spadella is out of scope for this paper. However, it is important to point out that the ventral part of the chaetognath gastrula is not neurectoderm, as the Reviewer writes. The neurectoderm is the lateral part of the gastrula (see orange dotted line on the Supplementary Fig. 3B), where cells proliferate to form the “lateral somata clusters”. They are pSMAD1/5-positive at least in their dorsal half. As development proceeds, these clusters fuse below the gut to form a ventral CNS. We changed the main text to make it more clear.

We write:

“Importantly, pSMAD1/5 signal is observed in the neuroectodermal regions that later contribute to lateral somata clusters, which will eventually fuse ventrally to form a CNS (Fig 9B-C’).”

In the last part of the Discussion (page 25), the authors discuss about the evolutionary scenario of "multiple independent centralizations of the nervous system across the bilaterian tree" and the necessary evidence to support this hypothesis. However, this hypothesis seems to be based on the assumption that diffused nervous system represents the ancestral condition in both protostome and deuterostome lineage, and that separate co-option of BMP-dependent DV patterning mechanism for the initial specification of neural ectoderm occurred multiple times for the evolution of CNS in different lineages. However, it seems equally possible that this co-option happened once in the common ancestor of bilaterians, and subsequently modified or lost in some lineages, resulting in a less centralized nervous system in some animals. I am curious how the authors could exclude this "single origin" scenario.

Response:

We agree that we cannot exclude multiple independent losses of the ancestral CNS across phyla, although we do favor the “simple urbilaterian” hypothesis. But thanks for pointing this out. We modified the concluding paragraph to include the “single origin” scenario.

Other minor points:

1. Fig 1 and its legend; please note that in 1C, it was not mentioned explicitly the red line indicates the directive axis and the blue dots indicate pSMAD1/5 activity; please confirm my interpretation. In addition, many abbreviations in 1D and 1E are not explained in the legend.

Response:

Yes, your interpretation is correct, we corrected the legend.

2. Page 11, the sentence "Among the different tissues only 9 genes were differentially regulated in all tissue samples (Fig 5C, S1 Table)"; This citation of figure should be "Fig 5D', and in Supplementary Table S1, I only saw 8 gene listed on the page "ov_all_tissues" and the number indicating "#DEGs present in all tissues" is also listed as 8. Please confirm this number.

Response:

Corrected, thank you!

3. Page 11, the sentence "Seven of these 20 were transcription factors or co-factors (Fig 5D, S2 Table)", the correct figure citation should be "Fig 5E". Please confirm.

Response:

Corrected, thank you!

**Rev. 2:**

This manuscript presents a thorough dissection of relationship between BMP2/4 activity and neural cell types and development in the anemone Nematostella. This is a very timely topic because there have been prominent arguments that a role of the BMP2/4 pathway in repressing neurogenesis is ancestral for bilaterians (based on evidence from vertebrates and flies). In many other taxa BMP activity co-occurs with neurogenesis indicating that it does not repress it. Thus, the further examination of this question in Nematostella and a cubomedusan represent an important advance, largely because of the position of cnidarians as an outgroup to Bilateria. The data is generally high quality, and well-presented and the writing is clear. I have only minor comments.

Minor comments

Title, abstract, elsewhere: "side effect" is not quite right; it's vague about something that we have specific terms for. I think what they are arguing is that it is a convergent trait that evolved in two lineages where the nervous system became highly centralized. Even "secondary" or "independently derived" would be more precise. Here is a suggestion for implementing that in the title.

The anti-neural role of BMP signaling in some taxa is independently derived from its ancestral role in global dorsoventral patterning

Response:

We changed the title to “The anti-neural role of BMP signaling is a consequence of its ancestral function in dorsoventral patterning”

"contradicted by the variety of the degrees of centralization of the bilaterian nervous systems - from diffuse to being arranged into a varying number of nerve cords to fully centralized"

I think the "ganglial" level of NS organization is an important category to recognize—many groups have nervous systems where there are multiple large ganglia connected by relatively small numbers of neurons that would not be called a net or a cord.

Response:

Thank you, we agree. The text was modified as follows:

While the role of BMP signaling in regulating the DV patterning is clearly ancestral and, with few exceptions, present across all Bilateria (Mörsdorf et al., 2024), its strictly “anti-neural” function is contradicted by the variety of the degrees of centralization of the bilaterian nervous systems – from diffuse to being arranged into a varying number of ganglia or nerve cords to fully centralized (Martín-Durán & Hejnol, 2021; Moroz, 2009).

"While neurons of the rhopalia are not marked by αTub, many pSMAD1/5-positive cells in the area between the stalk and the upper lens eye are likely to be neurons based on their location and cell morphology. pSMAD1/5-positive cells in this area exhibit a high cytoplasm to nucleus ratio (Fig 7J, white arrowheads)" This is not documented really at all. At the very least they would need to have much higher power images of these cells so the viewer could tell how much cytoplasm there was compared to nuclear volume. In addition, neural marker staining of the rhopalium would be useful to show that the staining is in a region of neurons. If they can't get these things, they should rephrase what these data imply, to something like "The location of these pSMAD+ cells is consistent with at least some having neural identities."

Response:

Thank you, we agree. We did not find the antibodies, which in our hands would stain neurons in the rhopalium, but we added a higher magnification image of pSMAD1/5 staining of the rhopalium and finished the paragraph with the sentence you suggested. We also asked Prof. Anders Garm (University of Copenhagen), who is an expert in cubozoan nervous system, and Dr. Yulia Kraus (University of Moscow), who is an outstanding cnidarian morphologist, to help us interpret our images. Prof. Garm, without being asked to, immediately identified the cells, at which white arrowheads are pointing, as giant neurons.

Figure 7, panel L, indicates the yellow stain is Pha, but there is no indication of what this is? Phalloidin?

Response:

Thanks for noticing this. We have added the Pha label and updated the figure description.

Last figure, panel D: Is there enough known from Knabl 2024, and Watanabe 2014 to include planula and or embryo stages of Nematostella in this panel along with polyp? These would seem to be more apt comparisons to the embryonic events summarized for other taxa here.

Response:

Early neurogenic events in the *Nematostella* embryo are well described - not only in the papers the Reviewer mentioned but also in many others from the Rentzsch, Layden, Martindale, and Technau groups. Our panel highlights the expression of neurogenic markers before detectable pSMAD1/5 activity at blastula stage and the largely radially symmetric arrangement of the diffuse nervous system along the directive axis at planula stage.

Also this panel, it's unclear to me how to understand the "mediolateral" patterning diagrams.. could they be put on a circular cross-section or is that not what they are trying to convey?

Also, on the top row of that panel, maybe put the "elav+" at the beginning (Danio) and/instead of the end?

Response:

We have removed the mediolateral gene expression diagrams since they are not further discussed in the paper. We have also removed the elav+ label.
