# Supplementary material for: The anti-neural role of BMP signaling is a consequence of its ancestral function in dorsoventral patterning
Source: PLoS Biol. 2026 May 21;24(5):e3003803. doi: 10.1371/journal.pbio.3003803 (PMC13193452; doi:10.1371/journal.pbio.3003803)
Supplement: Attachment — Submitted filename: Response to Reviewer.docx [file pbio.3003803.s017.docx]

Rev. 1:
In this revised manuscript, the authors have added a substantial amount of new data and images to address my previous comments. I have only a few minor points remaining that need to be corrected or clarified by the authors:

1. Page 11, Figure 4 legend; (K) should be (G-I). Please confirm.

Label corrected, (K) has been changed to (G-I).

2. Page 14, third line; (ts) should be (te). Please confirm.

Label corrected, (ts) has been changed to (te).

3. Page 14, 6th line, and Fig.6E; cannot see the "hollow yellow arrowhead" described here.

Thanks, "hollow yellow arrowhead" was removed from the text.

4. Page 14, 9th sentence (Fig. 6G-G", yellow arrowhead) should be (Fig. 6G-G", black outlined white arrowheads).

Corrected, "yellow arrowhead" has been changed to "white arrowheads". Black-outlined seems unnecessary, since there are no other arrowheads on the images

5. Page 23, 3rd line, legend of Figure 10; cannot see the "red arrowheads" on Fig. C or C'. Please confirm.

Corrected, the color of the arrowhead has been changed to red on the Figure.

We thank the Reviewer for noticing all these errors!
